# microRNAs regulate TAL1 expression in T-cell acute lymphoblastic leukemia

**DOI:** 10.18632/oncotarget.6987

**Published:** 2016-01-23

**Authors:** Nádia C. Correia, Alice Melão, Vanda Póvoa, Leonor Sarmento, Marta Gómez de Cedrón, Marcos Malumbres, Francisco J. Enguita, João T. Barata

**Affiliations:** ^1^ Instituto de Medicina Molecular, Faculdade de Medicina, Universidade de Lisboa, Lisboa, Portugal; ^2^ Cell Division and Cancer Group, Spanish National Cancer Research Centre (CNIO), Madrid, Spain

**Keywords:** T-cell acute lymphoblastic leukemia, T-ALL, TAL1, microRNAs, post-transcriptional regulation

## Abstract

The transcription factor TAL1 is a proto-oncogene whose aberrant expression in committed T-cell precursors is associated with the development of T-cell acute lymphoblastic leukemia (T-ALL). The mechanisms leading to aberrant activation of *TAL1* in T-ALL patients who lack chromosomal rearrangements involving the *TAL1* locus remain largely unknown. We hypothesized that TAL1 levels decrease during normal T-cell development at least in part due to miRNA-dependent silencing, in which case TAL1 over-expression in some T-ALL cases could be the consequence of deregulated miRNA expression. By performing computational prediction of miRNAs that bind to the human *TAL1* mRNA we compiled a list of miRNAs that are candidates to regulate *TAL1*. Using a luciferase reporter system and mutagenesis assays we confirmed the miRNA-TAL1 mRNA interactions and selected candidate miRNAs: miR-101, miR-520d-5p, miR-140-5p, miR-448 and miR-485-5p. Over-expression of these microRNAs in different T-ALL cell lines consistently resulted in the down-regulation of TAL1 protein. In accordance, inhibition of miR-101 and miR-520d-5p promoted TAL1 protein expression. Importantly, we found that miR-101, miR-140-5p, miR-448 and miR-485-5p were down-regulated in T-ALL patient specimens and T-ALL cell lines. Our results show for the first time the existence of epigenetic regulation of TAL1 by specific miRNAs which may contribute, at least in part, to the ectopic expression of TAL1 in some T-ALL cases.

## INTRODUCTION

In physiologic conditions, the basic helix-loop-helix transcription factor TAL1 is necessary for hematopoietic commitment [1-3], being expressed very early on in hematopoietic differentiation and silenced during normal T-cell lymphopoiesis from the early thymic progenitor stage onwards [4]. However, TAL1 is ectopically expressed in a majority of childhood T-cell acute lymphoblastic leukemia (T-ALL) cases, with increased *TAL1* transcript levels found in more than 60% of T-ALL patients [5, 6] even in the absence of obvious genetic alterations in the gene locus.

TAL1, as other T-cell oncogenes (e.g. HOX11 and LMO2), is commonly found mono or biallelically expressed in T-ALL patient blasts [7]. Either direct *cis*-activating mechanisms are involved in the gene monoallelic activation or the biallelic expression can be explained by the disruption of *trans*-acting mechanisms that normally down-regulate *TAL1* during T-cell development [7]. The most frequent chromosomal alteration involving the *TAL1* locus is the micro-deletion that fuses the *TAL1* coding region to the *SIL* regulatory elements, producing the *SIL-TAL1* fusion gene. This alteration occurs in 9–25% of childhood T-ALLs driving the aberrant monoallelic expression of TAL1 [8]. Recently, it was shown that aberrant expression of TAL1 can result from the formation of cellular context-dependent chromatin loops that mediate *cis*-activation of the *TAL1* locus [9, 10]. Additionally, elegant studies have revealed that micro-insertional mutating events occurring in heterozigosity upstream of the *TAL1* promoter can lead to monoallelic activation of *TAL1* [11, 12]. The exact frequency of TAL1-positive T-ALL cases due to these newly identified events remains to be determined, and thus a fraction of cases with TAL1 monoallelic aberrant expression, as well as those with ectopic biallelic activation, remain to be explained. In this context, regulation by non-coding RNAs (ncRNAs), such as microRNAs (miRNAs) and long non-coding RNAs (lncRNAs) [13] has not yet been thoroughly explored as a possible mechanism of epigenetic regulation of TAL1 expression in physiologic conditions or in malignancy.

MicroRNAs, the most comprehensively studied family of ncRNAs, are small (19-22 nt long), single stranded RNAs involved in post-transcriptional control of gene expression [14-16]. MicroRNAs target protein-coding genes through sequence-specific binding mainly to the 3′-untranslated region (3′UTR) of target messenger RNAs, which in mammals leads mostly to translational repression of the target gene [17]. Several studies [18-21] contributed to the notion that specific miRNA gene expression signatures are associated with particular B- and T-ALL oncogenetic subgroups. The participation of miRNA genes in T-ALL, individually or within a network, has been explored and specific miRNAs have been implicated in T-ALL pathogenesis [22-26]. Importantly, oncogenes with pivotal roles in the pathogenesis of T-ALL (such as TAL1 [27, 28] and NOTCH1 [29, 30]) have been associated with deregulated miRNA networks in this context.

In the present work, we sought to get further insight into the mechanisms that are involved in aberrant expression of TAL1 in T-ALL and particularly to understand if this process involves miRNAs. We hypothesized that TAL1 levels decrease during normal T-cell development at least in part due to miRNA-dependent down-regulation, in which case TAL1 over-expression in some T-ALL cases should be the consequence of deregulated miRNA expression.

## RESULTS

To investigate the existence of post-transcriptional regulation of *TAL1* by miRNAs we performed computational prediction of miRNAs that bind to *TAL1* 3′UTR. Computational algorithms have been the major driving force in predicting miRNA targets [31]. Several web-based bioinformatics tools were used to perform the preliminary identification of putative regulators of TAL1, as detailed in the Methods. The TAL1 3′UTR in humans has around 3.4kb (NM_003189) and harbors a high number of possible miRNA Recognizing Elements (MRE) distributed through the entire sequence (Supplementary Figure 1). From this initial analysis we compiled a list of 90 candidate miRNAs that might regulate TAL1 mRNA (Supplementary Table 1).

Next, we rationally narrowed the list down using the following criteria: a) miRNAs under-expressed in TAL or LMO overexpressing cases (TAL/LMO cytogenetic subgroup, based on primary T-ALL gene expression profiling [23]); b) concomitant identification of LMO2 as putative target, given the frequent aberrant co-expression of both TAL1 and LMO2; c) more than one predicted target site in the 3′UTR of the TAL1 mRNA and/or 8mer (or 9mer) type of seed paring; d) identification of the miRNA as regulator of *TAL1* expression by at least two different algorithms. Any miRNA predicted to fulfill at least one of these criteria was included for further testing. In this way, we narrowed down the list of putative miRNAs targeting *TAL1* to 39 (Supplementary Table 2).

**Figure 1:**
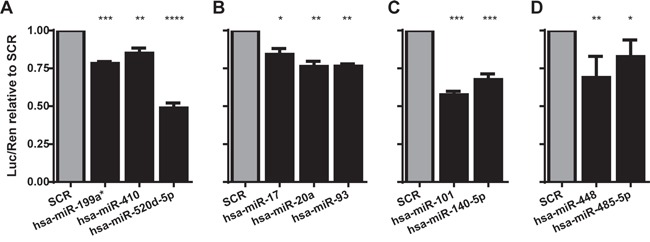
MicroRNAs identified as down-regulating TAL1 3′UTR reporter The ability of miRNAs to directly target TAL1 was evaluated using a reporter construct carrying the TAL1 3′UTR downstream of the luciferase gene. Results depict only the miRNAs that originated significant differences as compared to scramble sequence, to which all results have been normalized. miRNAs predicted to target TAL1 were ‘short-listed’ based on the following non-exclusive criteria: **A.** under-expressed in TAL1/LMO cytogenetic subgroup; **B.** predicted to also target LMO2; **C.** with more than one predicted target site and/or 8mer (or 9mer) type of seed paring; **D.** predicted to target TAL1 by at least two different prediction algorithms. The graphs represent at least two independent experiments with two replicates; statistical analysis was performed by One-way ANOVA (*p<0.05; **p<0.01; ***p<0.001).

**Figure 2:**
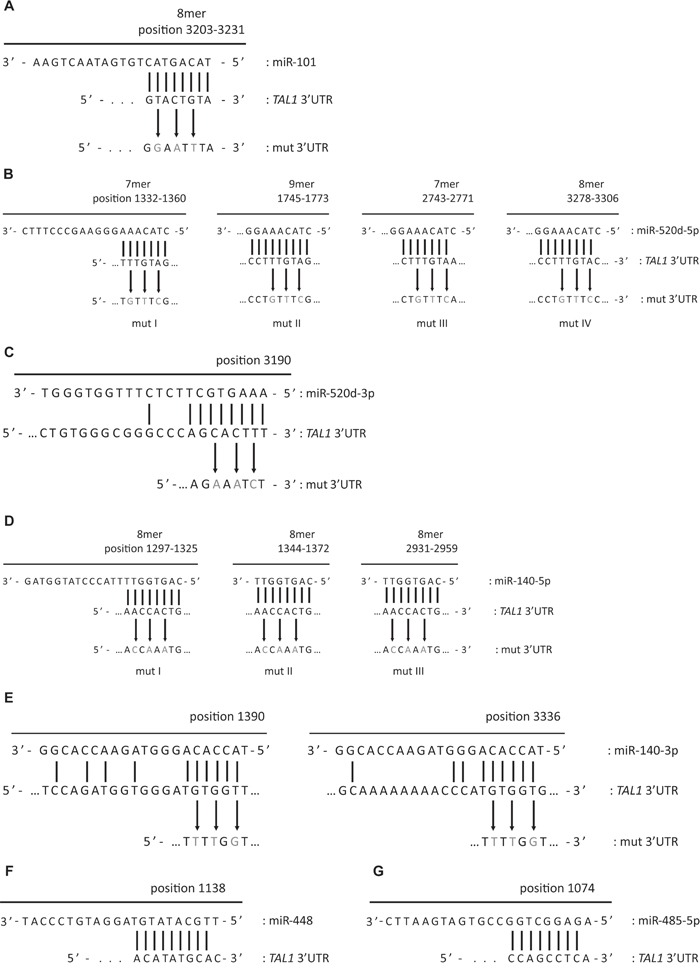
Schematic representation of microRNAs binding to TAL1 3′UTR and respective mutagenesis performed to disrupt miRNA seed binding Details of binding of **A.** miR-101; **B.** miR-520d-5p and **D.** miR-140-5p to TAL1 3′UTR are depicted according to DianaMicroT [71] target prediction algorithm. The miRNAs miR-520-3p **C.** and miR-140-3p **E.** are not predicted to bind to *TAL1* 3′UTR, nevertheless the putative MRE were mutated as depicted. The nucleotides altered in the mutagenesis assays are depicted in grey. **F.** miR-448 and **G.** miR-485-5p binding to *TAL1* 3′UTR details are depicted according to microRNA.org [68-70] target prediction algorithm.

In order to validate the candidate miRNA/TAL1 mRNA interaction, we transiently co-transfected 293T cells with a reporter plasmid coding the *TAL1* 3′UTR immediately downstream of the luciferase open reading frame (pLuc-TAL1-3′UTR), together with the candidate miRNAs from the miR-Vec library [32, 33]. We then verified if the reporter expression was decreased when compared to the control scramble (SCR) sequence, which is indicative of the miRNA biological activity against TAL1 3′UTR (Figure 1A-1D). For further analysis we selected microRNAs that significantly lowered the luciferase expression in 25-50%: miR-101, miR-520d-5p, miR-140-5p, miR-448 and miR-485-5p (see Figure 2 for miRNA binding details). We excluded from further analysis the miR-Vec-20a, miR-Vec-17 and miR-Vec-93, since the miRNAs encoded belong to the oncogenic cluster miR-17-92 (miR-17-5p and miR-20a-5p) or to the same family (miR-93-5p). The cluster miR-17-92 is highly up-regulated in hematopoietic malignancies and has a clearly defined oncogenic function [34-36], which was not consistent with our hypothesis that the candidate miRs should act as tumor suppressor-like genes (by down-regulating TAL1). Moreover, we excluded miR-Vec-410 and miR-Vec-199a* due to the weak effect on luciferase expression.

**Figure 3:**
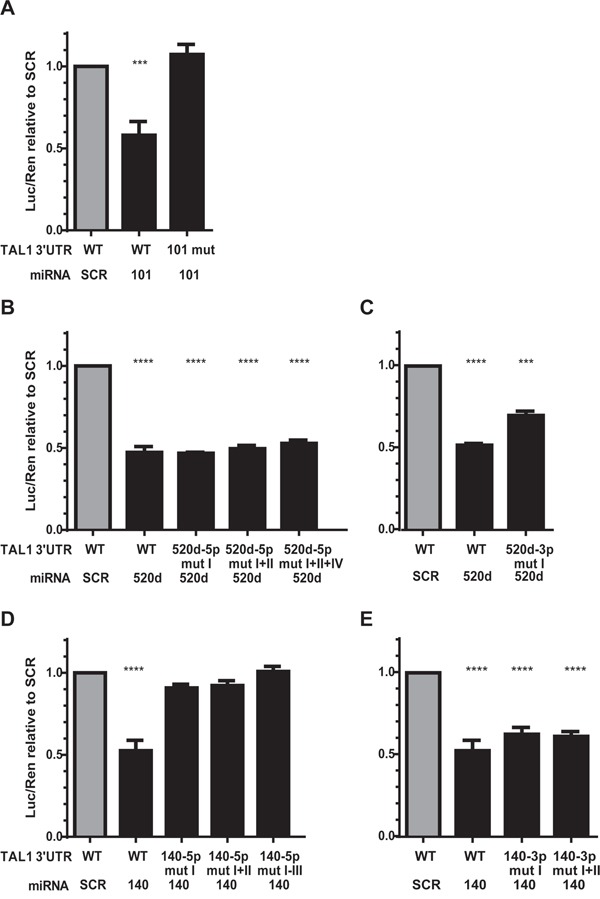
Effect of mutagenesis on microRNA-mediated repression of TAL1 3′UTR Luciferase activity of wild-type (WT) or mutant (mut) *TAL1* 3′UTRs in the presence of the corresponding miRNAs. Results were normalized to scramble (SCR) miRNA on WT *TAL1* 3′UTR: **A.** miR-101, **B, C.** miR-520d, and **D, E.** miR-140. The mutations were made in a cumulative manner, i.e. mut II was performed on the 3′UTR bearing already mut I and so on. The graphs represent 4 independent experiments with two replicates. Statistical analysis was performed by One-way ANOVA using the WT SCR condition as control condition. (***p<0.001; ****p<0.0001).

We then mutated the MRE in the 3′UTR of *TAL1* in order to disrupt the miRNA/mRNA binding (Figure 2) and re-evaluated the capacity of the respective miRNA to silence the reporter. Mutation of the only binding sequence for miR-101 in *TAL1* 3′UTR (Figure 2A) fully restored luciferase expression in the presence of the miR-Vec-101 (Figure 3A) demonstrating that the mutated sequence corresponds to the recognizing element of miR-101 in TAL1 3′UTR. The miR-520d-5p has four predicted binding sites in the 3′UTR (Figure 2B) and we were able to mutate three of them. The triple mutants in TAL1 3′UTR did not restore the luciferase expression in the presence of miR-520d (Figure 3B). The miR-520d precursor can give rise to two mature forms of the microRNA, the 5p from the 5′ arm of the hairpin and 3p from the 3′ arm of the hairpin, both expressed by miR-Vec vectors. The mutation of a putative binding site for miR-520d-3p in the 3′UTR (Figure 2C) increased the luciferase expression by 15% when compared with the non-mutated 3′UTR (Figure 3C), suggesting that the down-regulation of the reporter for miR-520d was partly dependent on the 3p form. Finally, the mutation of one of the three MREs for miR-140-5p (Figure 2D) was sufficient to restore luciferase expression (Figure 3D). This demonstrated that these elements are true recognition sites for miR-140-5p in the *TAL1* 3′UTR. Mutation of the two putative binding sites for miR-140-3p in the TAL1 3′UTR (Figure 2E) did not significantly affect reporter activity (Figure 3E), showing that the miR-140-3p is not the specimen responsible for the effect of miR-Vec-140 on the reporter expression.

**Figure 4:**
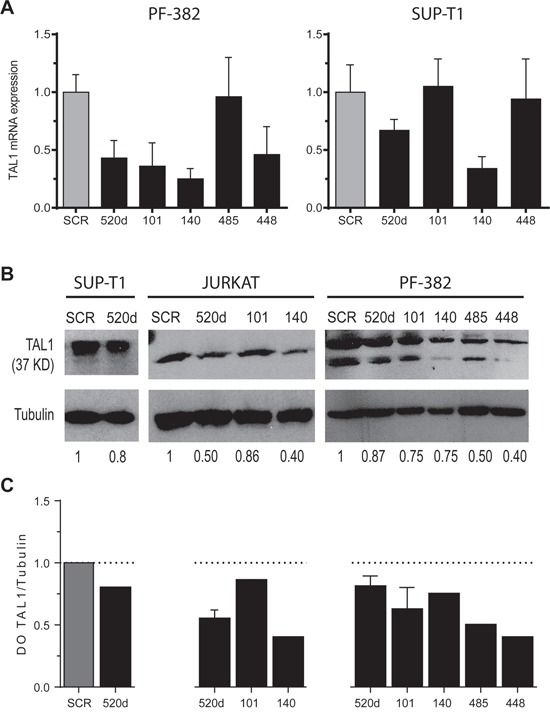
Ectopic expression of miR-520d, -101, -140, -485 and -448 down-regulates endogenous TAL1 mRNA and protein levels in T-ALL cell lines **A.** TAL1 transcript levels were analyzed by qPCR upon transfection of PF-382 and SUP-T1 cells with miR-Vec vectors. Values indicate the mean ± lower and upper limit of 3 technical replicates relatively to the scramble (SCR) transfection. **B.** TAL1 protein levels were evaluated by western blot analysis in T-ALL cell lines upon transfection with miR-Vec vectors. The relative TAL1 protein levels in T-ALL cell lines were normalized using α-Tubulin as loading control. **C.** Densitometric values (DO) of TAL1 levels (ratio to loading control) normalized to those measured in the presence of the respective scramble vector.

If a given mRNA is a true target of a specific miRNA, then modulation of the miRNA concentration should result in changes in the amount of protein encoded by the target gene. Thus, in order to evaluate the physiological importance of the miRNA/TAL1 mRNA pairs we over-expressed the candidate miRNAs in T-ALL cell lines that endogenously over-express TAL1, followed by evaluation of endogenous TAL1 mRNA and protein levels. Over-expression of miR-520d, miR-101, miR-140, miR-485 and miR-448 in different T-ALL cell lines resulted in down-regulation of TAL1 transcript (Figure 4A) and/or protein (Figure 4B-4C) expression levels in a range of 20-60%. This range is in accordance to the predicted effects of miRNAs in protein expression [37, 38] and it varies depending on the cell line and miRNA specimen. Down-regulation mediated by these miRNAs in the *TAL1* transcript was observed for miR-520d, miR-101, miR-140 and miR-448 in PF-382 cells and for miR-520d and miR-140 in SUP-T1 cells (Figure 4A). Why this heterogeneity occurs amongst T-ALL cell lines, and what it might inform on how TAL1 is regulated by miRNAs, requires further investigation. In any case, these results show that ectopic expression of the selected miRNAs can target TAL1 by affecting the mRNA stability and/or impairing the protein translation in the T-ALL cell lines analyzed.

**Figure 5:**
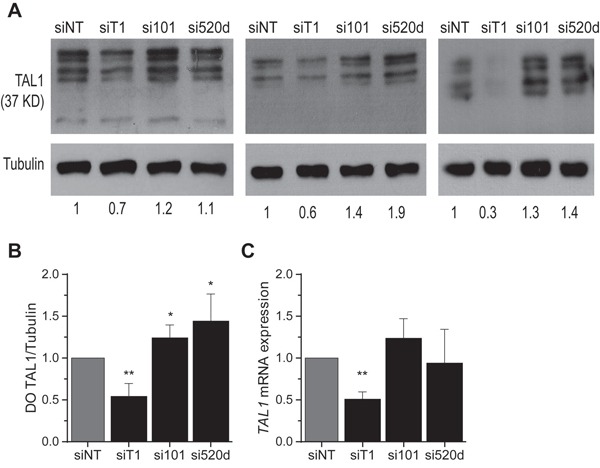
Inhibition of miR-520d-5p and miR-101 increases endogenous TAL1 protein levels in T-ALL cells **A.** Western blot and densitometric and analysis of TAL1 expression in CCRF-CEM cells upon nucleofection with microRNA inhibitors (si101 against hsa-miR-101 or si520d against hsa-miR-520d-5p), a siRNA against TAL1 (siT1) or a non-targeting siRNA control (siNT). α-Tubulin was used as loading control. Here are represented three independent nucleofection experiments. The numeric values depicted bellow represent the densitometric values normalized to the TAL1 expression in the siNT control for each experiment. **B.** Densitometric values (DO) of TAL1 expression of four independent nucleofection experiments were normalized to the α-Tubulin expression and compared to the control. **C.** *TAL1* transcript levels analysis by qPCR of the same three independent nucleofection experiments depicted in (A) (B, C) Values indicate the mean ± standard deviation relatively to the scramble nucleofection of independent nucleofection experiments and were analyzed using a Student's t-test (* p<0.05; **p<0.01).

To complement the previous experiment, we transfected cells with high endogenous miRNA levels and correspondingly low TAL1 expression, with antisense oligoribonucleotides (ASO) to inhibit the function of the endogenous mature miRNAs and evaluate their impact on TAL1 expression. We verified that inhibition of miR-520d-5p and miR-101 rescued endogenous TAL1 protein expression by 20 to 40% on average (Figure 5A-5B). This increase in protein expression was not always accompanied by a *TAL1* transcript increase (Figure 5C), in accordance with the previous experiments. Therefore, miR-520d-5p and miR-101 affect TAL1 mostly at the level of translation in T-ALL cells.

**Figure 6:**
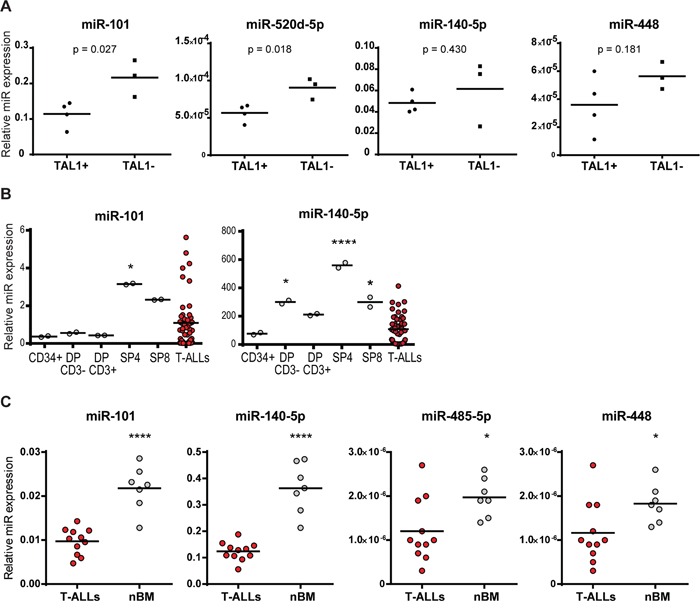
MicroRNA expression in T-ALL cell lines, patients and normal counterparts **A.** Expression of miR-101, miR-520d-5p, miR-140-5p and miR-448 was determined by qRT-PCR and normalized to SNORD38B expression in TAL1-positive (SUP-T1, CCRF-CEM, TAIL7, PF-382) and TAL1-negative (HPB-ALL, P12-ICHIKAWA, TALL-1) T-ALL human cell lines. Groups were compared using a 2-tailed Student's t-test. **B, C.** Expression of indicated miRNAs was analyzed using publicly available datasets. (B) MiRNA expression in T-ALL patients was compared to normal thymic populations (DP – double positive; SP4 – single positive CD4; SP8 – single positive CD8 T-cells). Data collected from [23]. (C) MiRNA expression in T-ALL patients was compared to normal bone marrow samples (nBM). Data collected from [20]. Statistical analysis was performed by One-way ANOVA (*p<0.05; ****p<0.0001).

Subsequently, we compared the expression of the miRNAs between TAL1-positive and -negative T-ALL cell lines and verified that they were (miR-101, 520d-5p) or tended to be (miR-140-5p and miR-448; not reaching statistical significance), more expressed in the TAL1-negative cell lines. These observations favor our hypothesis that TAL1 over-expression in some T-ALL cases may result from, or be potentiated by, decreased expression of specific miRNAs (Figure 6A). This idea was further strengthened by the fact that these miRNA genes were expressed in normal human thymic populations and that their expression was modulated during T-cell differentiation (Figure 6B).

We then reasoned that if these miRNAs play a role in TAL1 over-expression in T-ALL their levels should be decreased in primary leukemia cells as compared to normal developing T-cells. In agreement, we found that miR-101 and miR-140-5p were less expressed in T-ALL patient samples than in more differentiated thymocytes (Figure 6B). This is in accordance with the fact that TAL1 mRNA is not detected beyond the early thymic progenitor stage [4]. Moreover, T-ALL patient samples expressed lower levels of miR-101, miR-140-5p, miR-448 and miR-485-5p as compared to normal bone marrow cells (Figure 6C), which still express TAL1 (data not shown). These data provide support to the notion that the identified miRNAs may act as physiological upstream negative regulators of TAL1 that are aberrantly down-regulated in T-ALL, thereby contributing to TAL1 overexpression.

## DISCUSSION

While it is now known that TAL1 regulates the expression of numerous miRNA genes [27, 28], several observations led us to hypothesize that upstream miRNA networks could be involved in potentiating TAL1 overexpression in T-ALL. First, more than 60% of T-ALL patients display aberrantly high levels of *TAL1* transcript but only roughly half display chromosomal translocations or DNA rearrangements known to activate *TAL1* transcription [5]. Second, TAL1 is a putative target of several miRNAs that are up-regulated in hematopoietic stem cells, such as hsa-miR-17-5p, hsa-miR-197, hsa-miR-106 and hsa-miR-20 [39], and of some that are down-regulated in differentiated megakaryocytes, such as hsa-miR-106 and hsa-miR-20 [40], suggesting that miRNAs might regulate TAL1 at different stages of hematopoietic development. Finally, the LIM only protein LMO2, a TAL1 co-factor, was shown to be regulated by a microRNA, miR-223, namely during erythroid differentiation [41]. The majority of T-ALL patients with LMO ectopic expression also overexpress TAL1 [6] and both transcription factors cooperate to induce leukemia in transgenic mice [42]. These facts contributed to our speculation that, similar to LMO2, TAL1 could be regulated by miRNAs during normal hematopoiesis, in such way that TAL1 levels decrease during normal T-cell development at least in part due to miRNA-dependent down-regulation. In this case, TAL1 over-expression in some T-ALL cases could result from down-regulation of critical miRNA genes in the context of the disease. In other words, these miRNAs would have a tumor suppressor-like role by keeping in check TAL1. Interestingly, miR-101 [20, 43-52], miR-140-5p [53-56], miR-520-5p [57], and miR-485-5p [58-60], are all reported as putative tumor suppressors in different cancers.

The tumor suppressive function of miR-101 in inhibiting cell proliferation, migration, invasion and tumor growth, has been demonstrated in prostate [43], bladder [44], gastric [45, 48], and renal cell [49] carcinoma, pancreatic ductal adenocarcinoma [50] and melanoma [52], mainly due to targeting of the histone methyltransferase EZH2. In the context of hematopoietic malignancies, miR-101 was found down-regulated in samples of Burkitt lymphoma [46] and pediatric B-ALL patients [20, 51]. Adult T-cell leukemia/lymphoma patient cells have increased expression of EZH2 that is inversely correlated with the expression of miR-101 [47]. However, deleterious mutations in *EZH2* have been found in T-ALL patients (mainly associated with immature and not more differentiated cases), suggestive of a tumor suppressor role in T-cells. In our work, we showed that TAL1 is a direct target of miR-101 in T-ALL cell lines, in accordance with studies indicating a possible tumor suppressor role of miR-101 has in hematological malignancies. Given that our previous findings about TAL1-transcriptionally regulated miRNA expression [27] did not recognize miR-101 as a possible transcriptional TAL1 target, the low expression of the miRNA found in primary T-ALL samples and cell lines foresees a tumor-suppressive role for miR-101 in T-cell transformation, which might be in part mediated through down-regulation of TAL1 rather than EZH2. The miR-140-5p has also been associated with suppression of tumorigenesis in osteosarcoma and in colon [53], breast [54], lung [56] and hepatocellular [55] carcinoma. So far, the direct validated targets of this miRNA include stem cell self-renewal regulator SOX2 in breast cancer [54], TGFBR1 and FGF9 in hepatocellular carcinoma [55], SOX9 and ALDH1 in ductal carcinoma in situ [61] and IGF1R in lung cancer [56]. In addition, predicted targets for miR-101 besides *TAL1* include *MCL1* and *RUNX1*, being the latest a downstream TAL1 target and part of the TAL1+ gene signature described for T-ALL patients. *VEGF* and *HDAC4* are miR-140-5p predicted targets. Whether and how regulation of any of these genes by miR-101 and miR-140-5p is biologically relevant for thymocyte development and leukemogenesis, and may complement effects on TAL1, has not been addressed so far.

Given the relatively mild effects of each miRNA on TAL1 protein expression, which are in accordance to what is described for microRNA post-transcriptional regulation [37, 38], we do not foresee that their deregulated expression can fully justify the high levels of *TAL1* ectopic expression observed in T-ALL patients, unless it occurred in a coordinated fashion involving several TAL1-targeting miRNA genes as a consequence of a putative common upstream event. On the other hand, the fact that miR-140-5p is up-regulated in the different thymocyte subpopulations as compared to CD34+ cells may suggest that miR-140-5p could be part of the regulatory network that prevents TAL1 expression in normal committed thymic progenitors. In fact, the microRNA genes we identified, and others that remain to be, may partake in preventing TAL1 expression at different stages of T-cell development. For instance, miR-101 may be involved in TAL1 inhibition especially within SP thymocytes (where it is most highly expressed). In this way, different sets of microRNA genes could be involved, probably in coordination with other epigenetic mechanisms, in the continued silencing of TAL1 throughout normal T-cell development.

Also of potential relevance to the regulation of TAL1 by microRNAs in the context of T-ALL is the knowledge that mRNA transcripts in cancer cells frequently display shorter 3′UTRs then those in normal cells [62]. Putative poly-adenylation sites are present in the 3427 nt-long *TAL1* 3′UTR: two close AAUAAA poly-adenylation sites at positions 3052 and 3413 and one alternative AAGAAA poly-adenylation site at 1300, raising the possibility that shorter transcripts could exist in T-ALL cells that would exclude the MRE of some of the targeting miRNAs, consequently rendering TAL1 more ‘resistant’ to miR-mediated downregulation and thereby promoting TAL1 overexpression. While we have not explored this possibility in the current manuscript, we note that even if such transcripts prevail in T-ALL cases, the most upstream canonical poly-adenylation site would only protect the MRE corresponding to miR-520-3p, miR-140-3p (both of which we showed to have no effect on reporter expression recovery upon mutagenesis assays) and the 4th MRE for miR-520-5p, which we showed that, together other two MREs for miR-520-5p, is not sufficient to recover the reporter expression. In other words, the canonical poly-A sites are not predicted to protect *TAL1* mRNA from the microRNAs analyzed in this study.

Given the evidence we presented for TAL1 post-transcriptional regulation by microRNAs we speculate that TAL1 ectopic expression in T-ALL may, in some cases, be amplified by abnormal down-regulation of miRNAs targeting TAL1. This hypothesis implicates, once again, that miRNAs are physiologically involved in TAL1 regulation during normal development. Upon commitment to specific hematopoietic lineages the regulation of *TAL1* expression involves enhancer/promoter interactions, epigenetic alterations and trans-acting mechanisms, resulting in *TAL1* silencing in the lymphoid lineage. A possibility is that modest mRNA destabilization perpetuated by the miRNAs quickly yields substantial repression of protein output after transcription of the mRNA ceases. A similar mode of action has been described for other miRNAs in other physiological conditions [63]. In this model, depending on the threshold level for protein function, the mRNA decay rate, and the protein decay rate, modest miRNA-mediated repression can lead to substantially reduced protein and a much more rapid transition to the off state. If the miRNA also mediates translational repression, the transition to the off state is further accelerated [63]. If we consider that in a pre-leukemic stage the normal mechanisms that lead to *TAL1* transcriptional silencing are significantly decreased or disrupted, a concomitant down-regulation of miRNA-*TAL1* interactions could have a considerable positive impact on TAL1 protein levels and consequently on progression to overt leukemia. Evidently, a critical question that warrants investigation relates to the upstream mechanisms that lead to decreased expression of TAL1-regulating microRNAs in T-ALL.

## MATERIALS AND METHODS

### MicroRNA nomenclature and annotation

Official nomenclature and sequence annotation for the relevant microRNAs is based on the miRBase version 21 (2014) [64]. We listed the microRNA sequences relevant for this study in Supplementary Table 3. Regarding the miRNAs of interest, the only nomenclature difference from the miR-Vec library [33] refers to miR-101, which is currently named miR-101-3p according to miRBase version 21 [65]. The original nomenclature of the miR-Vec library vectors was kept [33].

### RNA extraction, RT-PCR and quantitative-PCR

RNA was extracted using TRIZOL (Life Technologies Corporation) according to the manufacturer's instructions. Amounts ranging from 100-1000 ng of total RNA were reverse transcribed using miRCURY LNA™ Universal RT kit (Exiqon). Real time PCR was performed with commercially available LNA-based primers (Exiqon) for mature microRNA detection with SYBRGreen (Exiqon) in a ViiA7 Real-Time PCR System (Life Technologies). Relative expression of the microRNAs was normalized to *SNORD38B* expression using the ddCt method.

### Computational prediction of TAL1 3′UTR targeting by microRNAs

PicTar (4-way) [66], TargetScanS release 4.2 [67], miRBase [64], microRNA.org [68-70], DIANA-microT algorithm V3.0 [71], miRDB [72] and StarBase [73] web-based bioinformatics tools were used, at the beginning of the study, to perform the identification of putative regulators of TAL1 and compiling the list of miRNAs that potentially regulate TAL1 transcript.

### Luciferase activity assays

A commercially available reporter plasmid coding for firefly and renilla luciferases and with *TAL1* 3′UTR (GeneCopoeia Inc) downstream of the luciferase open reading frame (pLuc-TAL1-3′UTR) was used. The miR-Vec vectors [32, 33], express the stem loop sequence (pre-miR) and were co-transfected into 293T cells. All the vectors used in this study were sequenced and the pre-miR sequences verified. All the miRNAs tested are listed in Supplementary Table 2. Briefly, 1.5×10^5^ 293T cells were cultured for 24h, and transfected with a mixture of 2μl Lipofectamine 2000 (Life Technologies), 200μl of OPTIMEM medium (GIBCO), 100 ng of the reporter vector and 500 ng of the miRNA-expressing vector. After 24h, cells were lysed and processed according to the Dual-Luciferase Reporter Assay System (Promega). The firefly luciferase and renilla luciferase activity was measured in an Infinite M200 plate reader (Tecan). For the firefly activity calculations, the values were normalized to renilla luminescence and the average of the two technical replicates was calculated. The values of at least three independent transfection experiments were normalized to the measurements of the corresponding scramble transfection.

### Site directed mutagenesis

To point-mutate the TAL1 3′UTR in the pLuc-TAL1-3′UTR vector, a PCR-based commercial kit was used according to the manufacturer instructions - QuikChange II XL site-directed mutagenesis (Agilent). All mutations were confirmed by sanger-sequencing. When more than one miRNA target site was possible for a given miRNA, the mutations were sequentially performed and named from the most upstream to the most downstream. The primers used in the site-directed mutagenesis are depicted in Supplementary Table 4.

### Cell lines

The human T-ALL cell lines were maintained in RPMI medium (GIBCO) supplemented with 10% FBS and split every 2-3 days. 293T cells were maintained in DMEM medium (GIBCO) supplemented with 10% FBS and split every 2 days. Cells were cultured at 37°C with 5% CO2. At the indicated time points, the cells were harvested and processed as indicated for RNA and protein extraction.

### Electroporation of miR-Vec vectors in T-ALL cell lines

Cell lines were transiently transfected with the corresponding miR-Vec vectors or scramble control (miR-Vec-SCR). To circumvent the difficulty imposed by low efficiency of transfection on the T-ALL cell lines, we co-transfected each miR-Vec with a GFP expressing vector (pMax, Lonza) and cells were sorted to enrich for high GFP+ populations. A total of 30μg of DNA (9μg of pMax and 21μg of miR-Vec) were added to 10^7^ T-ALL cells in the appropriate volume of pre-warmed RPMI-10 medium (without antibiotics). Electroporation was performed using Gene Pulser II in 4 cm–gap cuvettes (Bio-Rad), with the parameters depicted in Supplementary Table 5. After 24h, cells were sorted to obtain the GFP-expressing cells using a FACSAria III (BD Biosciences) and were collected for RNA and/or protein extraction 48h later.

### Nucleofection of T-ALL cells

Nucleofection of CCRF-CEM was performed using the Amaxa Nucleofector II (Lonza) with the X-001 program, according to the manufacturer's instructions. For miR-101 or miR-520d-5p knockdown, 2μM of miRCURY LNA™ microRNA Inhibitors (Exiqon) and non-targeting control were used. After nucleofection, the cells were cultured for 48h in RPMI-10 medium.

### Immunoblot

Cell lysates were prepared as described [74] using the following antibodies: α-Tubulin (Sigma, Clone DM 1A) and TAL1 (Millipore, clone BTL73). Densitometry analysis was performed using Adobe Photoshop CS5 Extended software. Each band was analyzed with a constant frame and normalized to the respective loading control. Densitometry values are expressed in arbitrary units.

### Statistical analysis

Statistical differences between mean values were evaluated using 2-tailed Student's t-test or One-way ANOVA, as appropriate. Significance was set for P<0.05. All analyses were performed using GraphPad Prism 6.0 (GraphPad Software).

## SUPPLEMENTARY FIGURE AND TABLES




