# Supplementary material for: microRNAs regulate TAL1 expression in T-cell acute lymphoblastic leukemia
Source: Oncotarget. 2016 Jan 23;7(7):8268–81. doi: 10.18632/oncotarget.6987 (PMC4884991; doi:10.18632/oncotarget.6987)
Supplement: Supplementary file 1 [file oncotarget-07-8268-s001.pdf]

SUPPLEMENTARY FIGURE AND TABLES

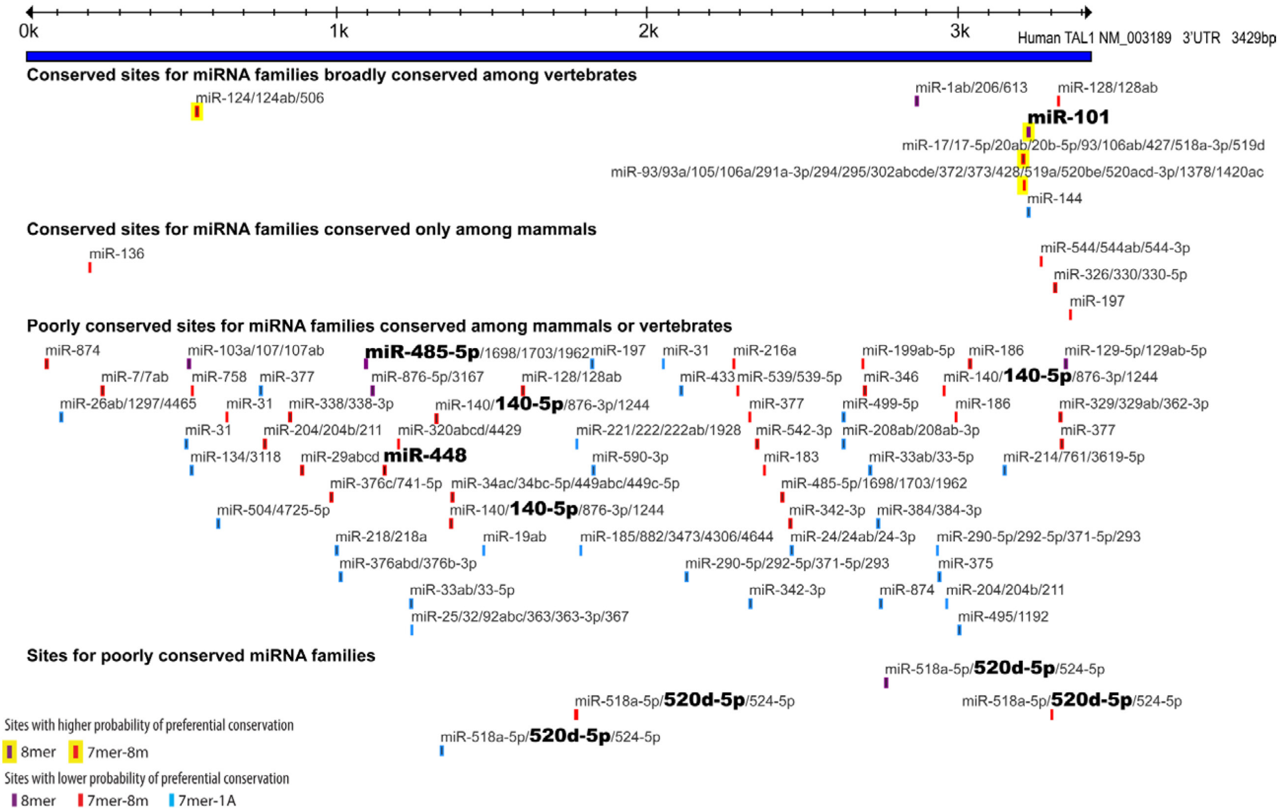

Supplementary Figure S1: Schematic representation of microRNA binding to the TAL1 3'UTR obtained with the target prediction algorithm TargetScanS release 4.2 [65]. The microRNAs selected for further analysis are highlighted in bold.

Supplementary Table S1: Computational prediction of TAL1 3'UTR targeting by microRNAs

See Supplementary File 1

Supplementary Table S2: List of microRNAs selected for testing in luciferase reporter assays, after the indicated criteria (detailed below and in the main text) were applied to the miRs listed in Supplementary Table 1

Criteria	miR-Vec tested
a) miRNAs under-expressed in TAL1/LMO cytogenetic subgroup	hsa-miR-1, hsa-miR-196, hsa-miR-199a*, hsa-miR-211, hsa-miR-302a-d, hsa-miR-34a, hsa-miR-373, hsa-miR-410, hsa-miR-506, hsa-miR-520c-3p, hsa-miR-520d-5p, hsa-miR-520e
b) miRNAs predicted to also target LMO2	hsa-miR-106a, hsa-miR-106b, hsa-miR-17, hsa-miR-186, hsa-miR-20a, hsa-miR-374a, hsa-miR-519d, hsa-miR-93
c) miRNAs with more than one predicted target site and/or 8mer (or 9mer) type of seed paring	hsa-miR-101, hsa-miR-140-5p, hsa-miR-206, hsa-miR-409-3p, hsa-miR-516b, hsa-miR-524-5p, hsa-miR-128, hsa-miR-377
d) miRNA predicted to target TAL1 by at least two different algorithms	hsa-miR-144, hsa-miR-197, hsa-miR-129-5p, hsa-miR-136, hsa-miR-204, hsa-miR-34c-5p, hsa-miR-448, hsa-miR-449a, hsa-miR-485-5p, hsa-miR-486-3p, hsa-miR-449a/b
**Genes showing up in at least one of the above criteria (tested in luciferase reporter assays)**	hsa-miR-1, hsa-miR-101, hsa-miR-106a, hsa-miR-106b, hsa-miR-128, hsa-miR-129-5p, hsa-miR-136, hsa-miR-140-5p, hsa-miR-144, hsa-miR-17, hsa-miR-186, hsa-miR-196, hsa-miR-197, hsa-miR-199a*, hsa-miR-20a, hsa-miR-204, hsa-miR-206, hsa-miR-211, hsa-miR-302a-d, hsa-miR-34a, hsa-miR-34c-5p, hsa-miR-373, hsa-miR-374a, hsa-miR-377, hsa-miR-409-3p, hsa-miR-410, hsa-miR-448, hsa-miR-449a, hsa-miR-449b, hsa-miR-485-5p, hsa-miR-486-3p, hsa-miR-506, hsa-miR-516b, hsa-miR-519d, hsa-miR-520c-3p, hsa-miR-520d-5p, hsa-miR-520e, hsa-miR-524-5p, hsa-miR-93

Supplementary Table S3: List of microRNAs relevant for this study and their sequence

miRNA name (v21)	Sequence
hsa-miR-101-3p	UACAGUACUGUGAUAACUGAA
hsa-miR-140-5p	CAGUGGUUUUACCCUAUGGUAG
hsa-miR-140-3p	UACCACAGGGUAGAACCACGG
hsa-miR-520-5p	CUACAAAGGGAAGCCCUUUC
hsa-miR-520-3p	AAAGUGCUUCUCUUUGGUGGGU
hsa-miR-448	UUGCAUAUGUAGGAUGUCCCAU
hsa-miR-485-5p	AGAGGCUGGCCGUGAUGAAUUC

Supplementary Table S4: List of primers used in site-directed mutagenesis

Mutation	Forward primer	Reverse primer
520-5p mut I	GTGAAGAATCCTTGTTTCGAATGAACC ACTGCCCCTTCATTGATTTCCTG	CAGGAAATCAATGAAGGGGCAGTGGTT CATTCGAAACAAGGATTCTTCAC
520-5p mut II	GGGCAACATTGTTCACCTGTTTCGCAC TCAGGCTCTCC	GGAGAGCCTGAGTGCGAAACAGGTGAA CAATGTTGCCC
520-5p mut III	GGGCAAGTCTTTAGGTCTGTTTCAGAACT AAAGAAGATCTG	CAGATCTTCTTTAGTTCTTACAAAGACCT AAAGACTTGCCCTTTCCTACC
520-5p mut IV	CAGGTACCTTGACCTGTTTCCAGCCCAGA GGCCAACAC	GTGTTGGCCTCTGGGCTGGAAACAGGTC AAGGTACCTG
520-3p mut I	CTGTGGGCGGGCCCAGAAATCTCCGT CAACGTTGTAC	GTACAACGTTGACGGAGATTTCTGGGCCC GCCCACAG
101 mut	GGCCCAGCACTTTCCGTCAACGTTGGAAT TTATGTGATGAATTGCG	CGCAATTCATCACATAAATTCCAACGTTGA CGGAAAGTGCTGGGCC
140-5p mut I	CCTTATCCTTCATCTTTTAAAGAAATAC CAAATGCAAGTCCTTTTGTAAAGTG	CACTTTACAAAAGGACTTGCATTTGGTA TTTCTTTAAAAGATGAAGGATAAGG
140-5p mut II	GAAGAATCCTTTTGTAGAATGACCAAAT GCCCCTTCATTGATTTCCTG	CAGGAAATCAATGAAGGGGCATTTGGTCA TTCTACAAAAGGATTCTTC
140-5p mut III	GAGAACAAAGATGACCATACCAAATGA AGGGAATCACATCTTTTAAGAC	GTCTTAAAAGATGTGATTCCCTTCATTTGG TATGGTCATCTTTGTTCTC
140-3p mut I	CAATCCAGATGGTGGGATTTTGGTTTCTT AAGGTGAGGCCTGTC	GACAGGCCTCACCTTAAGAAACCAAAAT CCCACCATCTGGATTG
140-3p mut II	GTGACTCTTTAGCAAAAAAAACCCATTTTG GGATGATGTGTATATATATG	CATATATATACACATCATCCCAAAATGGG TTTTTTTTGCTAAAGAGTCAC

The area of the miss-matching to the MRE element in the 3'UTR is shadowed in the forward primer.

Supplementary Table S5: Transfection conditions of T-ALL cell lines

Cell	Cell Nr	Volume	Volts	uF	pMAX	miR-Vec
SUP-T1	10M	350ul	350	750	9ug	21ug
JURKAT	10M	350ul	250	950	9ug	21ug
PF-382	10M	250ul	350	500	9ug	21ug
