# Supplementary material for: microRNAs regulate TAL1 expression in T-cell acute lymphoblastic leukemia
Source: Oncotarget. 2016 Jan 23;7(7):8268–81. doi: 10.18632/oncotarget.6987 (PMC4884991; doi:10.18632/oncotarget.6987)
Supplement: Supplementary file 2 [file oncotarget-07-8268-s002.docx]

**Supplementary Table 1** – Computational prediction of TAL1 3’UTR targeting by microRNAs.

| **miRNA** | **Program** | **Binding type** | **3’UTR position** | **Conservation** | **LMO2 targeting** | **DIANA-miRPath** |
| --- | --- | --- | --- | --- | --- | --- |
| hsa-miR-1-3p | miRDB(MirTarget2); TargetScanS; miRanda (microrna) | 8mer | 2815 | * |  | Adherent junction; glioma; thyroid cancer |
| hsa-miR-101-3p | miRDB(MirTarget2); TargetScanS; miRanda (microrna); PicTar (4-way); DianaMicroT | 8mer | 3174 | 9 * |  | MAPK signaling pathway; Colorectal cancer; Renal cell carcinoma |
| hsa-miR-103a-3p | miRanda (microrna) |  | 507 | * |  |  |
| hsa-miR-106a-5p | TargetScanS; PicTar (4-way); DianaMicroT | 7mer-m8 | 3156 | 8 | ✔ | MAPK signaling pathway; Chronic myeloid leukemia; TGF-beta signaling pathway |
| hsa-miR-106b-5p | TargetScanS; PicTar (4-way); DianaMicroT | 7mer-m8 | 3156 | 8 | ✔ | TGF-beta signaling pathway; Bladder cancer; MAPK signaling pathway |
| hsa-miR-107 | miRanda (microrna) |  | 507 | * |  |  |
| hsa-miR-1184 | miRDB(MirTarget2) |  | 523 |  |  |  |
| hsa-miR-124-3p | TargetScanS | 7mer-m8 | 545 |  |  | Gap junction; Melanogenesis; Axon guidance |
| hsa-miR-128-3p | TargetScanS | 7mer-m8 | 3271, 1595 |  |  |  |
| hsa-miR-1283 | StarBase |  |  |  |  |  |
| hsa-miR-1285-3p | miRDB(MirTarget2) |  | 2225, 2526 |  |  |  |
| hsa-miR-1291 | miRDB(MirTarget2) |  | 556 |  |  |  |
| hsa-miR-129-5p | miRDB(MirTarget2); miRanda (microrna) |  | 3332 | * |  |  |
| hsa-miR-136-5p | miRDB(MirTarget2); miRanda (microrna) |  | 187 | * |  | Nicotinate and nicotinamide metabolism; Butanoate metabolism; Starch and sucrose metabolism |
| hsa-miR-140-5p | miRDB(MirTarget2); miRanda (microrna); StarBase; DianaMicroT | 8mer | 1318, 1365, 2952 | * |  | adherent junction; Pancreatic cancer; Regulation of actin cytoskeleton |
| hsa-miR-144-3p | TargetScanS; PicTar (4-way); DianaMicroT | 7mer-1A | 3175 | 6 |  | Wnt signaling pathway; Maturity onset diabetes of the young; Reductive carboxylate cycle (CO2 fixation) |
| hsa-miR-148a-3p | miRanda (microrna) |  | 279 | * |  |  |
| hsa-miR-17-3p | DianaMicroT | 7mer-m8 | 3156 | 8 | ✔ | Bladder cancer; Axon guidance; Pancreatic cancer |
| hsa-miR-17-5p | TargetScanS; PicTar (4-way); DianaMicroT | 7mer-m8 |  |  | ✔ |  |
| hsa-miR-186-5p | miRDB(MirTarget2); miRanda (microrna; miRBase) |  | 2979, 3022 | * | ✔ | TGF-beta signaling pathway; Long-term potentiation; Wnt signaling pathway |
| hsa-miR-1915-3p | miRDB(MirTarget2) |  | 154, 647 |  |  |  |
| hsa-miR-196a-5p | miRanda (miRBase) |  |  |  |  | Cholera - Infection; ABC transporters - General; Novobiocin biosynthesis |
| hsa-miR-197-3p | TargetScanS; miRDB(MirTarget2); miRanda (microrna) | 7mer-m8 | 3309 | * |  |  |
| hsa-miR-1972 | miRDB(MirTarget2) |  | 305, 416, 2262 |  |  |  |
| hsa-miR-199a-3p | PicTar (4-way) |  | 3226 |  |  |  |
| hsa-miR-199a-5p | miRanda (microrna) |  | 2452, 2678 | * |  |  |
| hsa-miR-199b-5p | miRanda (microrna) |  | 2678 | * |  |  |
| hsa-miR-204-4p | PicTar (4-way); miRanda (microrna) |  | 3409 | * |  | Long-term potentiation; Chronic myeloid leukemia; Neurodegenerative Diseases |
| hsa-miR-206 | TargetScanS; miRDB(MirTarget2); miRanda (microrna) | 8mer | 2815-2821 | * |  | adherent junction; Focal Adhesion; Glioma |
| hsa-miR-20a-5p | TargetScanS; PicTar (4-way); DianaMicroT | 7mer-m8 | 3156 | 8 | ✔ | TGF-beta signaling pathway; Bladder cancer; pancreatic cancer |
| hsa-miR-20b-5p | TargetScanS; PicTar (4-way); DianaMicroT | 7mer-m8 | 3156 | 8 | ✔ | Bladder cancer; Axon guidance; pancreatic cancer |
| hsa-miR-211-5p | PicTar (4-way); miRanda (microrna; miRBase) |  | 3409 | * |  | Long-term potentiation; Chronic myeloid leukemia; Neurodegenerative Diseases |
| hsa-miR-216a-5p | miRanda (microrna) |  | 2263 | * |  |  |
| hsa-miR-302a-3p | TargetScanS; PicTar (4-way) | 7mer-m8 | 3155 |  |  | Chronic myeloid leukemia; TGF-beta signaling pathway; prostate cancer |
| hsa-miR-302b-3p | TargetScanS; PicTar (4-way) | 7mer-m8 | 3155 |  |  | Prostate cancer; TGF-beta signaling pathway; Chronic myeloid leukemia |
| hsa-miR-302c-3p | TargetScanS; PicTar (4-way) | 7mer-m8 | 3155 |  |  | Chronic myeloid leukemia; TGF-beta signaling pathway; Focal adhesion |
| hsa-miR-302d-3p | TargetScanS; PicTar (4-way) | 7mer-m8 | 3155 |  |  | Prostate cancer; TGF-beta signaling pathway; Chronic myeloid leukemia |
| hsa-miR-302e | TargetScanS |  |  |  |  | Ubiquitin mediated proteolysis; Huntington's disease; Amyotrophic lateral sclerosis (ALS) |
| hsa-miR-31-5p | TargetScanS |  | 2312 | * |  |  |
| hsa-miR-342-3p | miRanda (microrna) |  |  |  |  |  |
| hsa-miR-346 | miRanda (microrna) |  | 2682 | * |  |  |
| hsa-miR-34a-5p | miRanda (microrna); StarBase |  | 1355 | * |  |  |
| hsa-miR-34c-5p | miRanda (microrna); StarBase |  | 1356 | * |  |  |
| hsa-miR-372-3p | TargetScanS; PicTar (4-way) | 7mer-m8 | 3155 |  |  | Bladder cancer; melanoma; glioma |
| hsa-miR-373-3p | TargetScanS; PicTar (4-way) | 7mer-m8 | 3155 |  | ✔ | TGF-beta signaling pathway; Axon guidance; mTOR signaling pathway |
| hsa-miR-374a-5p | PicTar (4-way); miRanda (microrna) |  | 3361 | * | ✔ |  |
| hsa-miR-377-3p | DianaMicroT | various (3) | 728, 2305, 3309 | 0 |  | Wnt signaling pathway; Small cell lung cancer; TGF-beta signaling pathway; |
| hsa-miR-409-3p | miRDB(MirTarget2); DianaMicroT | 7mer(2);8mer | 1753, 2863, 2925 | 0-2-0 |  | Colorectal cancer; TGF-beta signaling pathway; adherent junction |
| hsa-miR-410-3p | miRanda (microrna) |  | 3359 | * | ✔ |  |
| hsa-miR-429 | miRanda (microrna) |  | 1030 | * |  |  |
| hsa-miR-433-3p | miRanda (microrna) |  | 2094 | * | ✔ |  |
| hsa-miR-448 | miRanda (microrna); StarBase |  | 1138 | * |  |  |
| hsa-miR-449a | miRanda (microrna); StarBase |  | 1355 | * |  |  |
| hsa-miR-449b-5p | miRanda (microrna); StarBase |  | 1355 | * |  |  |
| hsa-miR-485-5p | miRanda (microrna); StarBase |  | 1074 | * |  |  |
| hsa-miR-486-3p | StarBase |  | 3316 | * |  |  |
| hsa-miR-505-3p | miRanda (microrna) |  |  |  |  |  |
| hsa-miR-506-3p | TargetScanS; DianaMicroT | 7mer-m8 | 545 | 3 |  | Axon guidance; Melanogenesis; Acute myeloid leukemia |
| hsa-miR-516b-5p | DianaMicroT | 7mer;8mer(2) | 1376, 2271, 2556 | 2-1-0 |  | Circadian rhythm; Keratin sulfate biosynthesis; Notch signaling pathway |
| hsa-miR-519d-3p | TargetScanS; DianaMicroT | 7mer-m8 | 3156 | 8 | ✔ | Bladder cancer; pancreatic cancer; Axon guidance |
| hsa-miR-520a-3p | TargetScanS | 7mer-m8 | 3155 |  |  | Pancreatic cancer; Bladder cancer; Chronic myeloid leukemia |
| hsa-miR-520b | TargetScanS | 7mer-m8 | 3155 |  |  | Pancreatic cancer; Bladder cancer; glioma |
| hsa-miR-520c-3p | TargetScanS | 7mer-m8 | 3155 |  |  | Pancreatic cancer; Bladder cancer; glioma |
| hsa-miR-520d-5p | miRDB(MirTarget2); DianaMicroT | 7mer(2);8mer;9mer | 1332, 1745, 2743 , 3278 | 7-4-0-1 |  | Pancreatic cancer; Bladder cancer; TGF-beta signaling pathway |
| hsa-miR-520d-3p | TargetScanS | 7mer-m8 | 3155 |  |  | adherent junction; GnRH signaling pathway; MAPK signaling pathway |
| hsa-miR-520e | TargetScanS | 7mer-m8 | 3155 |  |  | Pancreatic cancer; Bladder cancer; glioma |
| hsa-miR-520f-3p | DianaMicroT | 7mer(1);8mer(2) | 2562, 3183 | 8-0 |  | TGF-beta signaling pathway; Focal adhesion; Wnt signaling pathway |
| hsa-miR-524-5p | miRDB(MirTarget2); StarBase; DianaMicroT | 7mer(2);8mer;9mer | 1766, 2764, 3299 | 7-4-0-1 |  | adherent junction; MAPK signaling pathway; GnRH signaling pathway |
| hsa-miR-539-5p | TargetScanS |  |  |  |  | Acute myeloid leukemia; TGF-beta signaling pathway; Chronic myeloid leukemia |
| hsa-miR-544a | TargetScanS; miRanda (microrna) | 7mer-m8 | 3214 | * |  |  |
| hsa-miR-545-3p | miRanda (microrna) |  | 949 |  |  | Regulation of actin cytoskeleton; Melanogenesis; Renal cell carcinoma |
| hsa-miR-548a-5p | DianaMicroT | 7mer;8mer pos1 | 197, 2463 | 5-11 |  | Ubiquitin mediated proteolysis; Colorectal cancer; Pancreatic cancer |
| hsa-miR-548b-5p | DianaMicroT | 7mer;8mer pos1 | 197, 2463 | 5-11 |  | TGF-beta signaling pathway; colorectal cancer; pancreatic cancer |
| hsa-miR-548c-5p | DianaMicroT | 7mer;8mer pos1 | 197, 2463 | 5-11 |  | TGF-beta signaling pathway; Ubiquitin mediated proteolysis; Colorectal cancer |
| hsa-miR-548d-5p | DianaMicroT | 7mer;8mer pos1 | 197, 2463 | 5-11 |  | Colorectal cancer; pancreatic cancer; TGF-beta signaling pathway |
| hsa-miR-548n | miRDB(MirTarget2) |  | 220, 2485 |  |  |  |
| hsa-miR-576-3p | miRDB(MirTarget2) |  | 2968 |  |  | Olfactory transduction; Adipocytokine signaling pathway; Long-term depression |
| hsa-miR-599 | miRanda (microrna) |  | 982 | * |  |  |
| hsa-miR-603 | DianaMicroT | 7mer pos1 | 2287 | 0 | ✔ | Axon guidance; adherent junction; Renal cell carcinoma |
| hsa-miR-604 | miRanda (microrna) |  | 887 |  |  | ErbB signaling pathway; Type II diabetes mellitus; Thiamine metabolism |
| hsa-miR-612 | miRDB(MirTarget2); DianaMicroT | 7mer (2); 9mer | 2204 , 2505, 3179 | 0-0-7 |  | Basal cell carcinoma; Wnt signaling pathway; Endometrial cancer |
| hsa-miR-613 | TargetScanS; miRanda (microrna) | 8mer | 2815 | * |  | mTOR signaling pathway; Dorso-ventral axis formation; adherent junction |
| hsa-miR-640 | miRDB(MirTarget2) |  | 1994 |  |  | Basal cell carcinoma; Melanogenesis; Renal cell carcinoma |
| hsa-miR-7-5p | miRanda (miRBase) |  |  |  |  | Axon guidance; ErbB signaling pathway; Insulin signaling pathway |
| hsa-miR-766-3p | DianaMicroT | 7mer; 9mer(2) | 263, 286, 2339 | 0 |  | Cell adhesion molecules; Type I diabetes mellitus; Glycerophospholipid metabolism |
| hsa-miR-874-3p | miRanda (microrna) |  | 2643 | * |  |  |
| hsa-miR-93-5p | TargetScanS; PicTar (4-way); DianaMicroT | 7mer-m8 | 3156 | 8* | ✔ |  |

##### Several web-based bioinformatics tools (PicTar (4-way), TargetScanS release 4.2, miRBase, microRNA.org, DIANA-microT algorithm, miRDB and StarBase) were used to perform the identification of putative regulators of TAL1.

##### MiRNAs nomenclature is based on the miRBase version 21 (2014). The putative microRNAs binding type, when defined, were listed according to the TargetScanS or DianaMicroT (for 9mer) as following: 7mer-m8 site comprises the seed plus a match to miRNA nucleotide 8 (miRNA nucleotides 2–8); 7mer-A1 site comprises the seed supplemented by an Adenine at target position 1; 8mer site that comprises a match to miRNA nucleotide 8, the miRNA seed and the A at position 1; and 9mer defines an exact match on the positions 1-10. The UTR position of the putative binding sites are depicted according to TargetScanS. The conservation score was listed according to DianaMicroT program prediction, when available: it depicts the number of species in which the binding nucleotides of this target site are conserved. We also listed the target sites of conserved microRNAs with good mirSVR score according to microRNA.org program. The mirSVR scores are based on a regression method for predicting the likelihood of target mRNA down-regulation from sequence and structure features in microRNA/mRNA predicted target sites. Additionally we also listed the microRNAs that are also predicted to target LMO2. Finally, we used the DIANA-miRPath tool to predict the main biological pathways where the putative targets of the microRNA are involved.
